# Perception of clinical teachers about their roles and current practice at affiliated hospitals of medical universities in China

**DOI:** 10.1080/10872981.2024.2325182

**Published:** 2024-03-11

**Authors:** Jinmeng Huang, Chunxia Huang, Jinmei Chen, Kaiyong Huang

**Affiliations:** a Educational Evaluation and Faculty Development Center, Guangxi Medical University, Nanning, Guangxi, China; b School of Foreign Languages, Guangxi Medical University, Nanning, Guangxi, China; c Department of Occupational Health and Environmental Health, School of Public Health, Guangxi Medical University, Nanning, Guangxi, China; d Guangxi Colleges and Universities Key Laboratory of Prevention and Control of Highly Prevalent Diseases, Guangxi Medical University, Nanning, Guangxi, China; e Guangxi Key Laboratory of Environment and Health Research, Guangxi Medical University, Nanning, Guangxi, China

**Keywords:** Clinical teacher, medical education, teacher role, perception, practice

## Abstract

*Phenomenon*: The increase in clinical and teaching workload has brought enormous pressure to clinical teachers. Clinical teachers play an extremely important role in the quality of higher medical education and the cultivation of medical talents. However, few studies have examined the attitudes and practices of clinical teachers regarding the role of teachers in China. This study aimed to investigate clinical teachers’ perceptions about their roles and current practices at affiliated hospitals of medical universities in China. *Approach*: Responses from 312 Chinese clinical teachers were included in the analyses. The data were collected using the questionnaires of perception and practice regarding the role of teachers which consisted of 12 items rated on a 5-point Likert scale, ranging from 1 (strongly disagree) to 5 (strongly agree), and scored by calculating the mean. The data were analyzed using the Statistical Package for Social Sciences, version 22.0 (IBM SPSS Corp). *Findings*: The mean score of perception of clinical teachers regarding the role of teachers was 4.51 (SD = 0.72), and the mean score of practice was 3.69 (SD = 1.17). Multivariable binary logistic regression model showed that undertaking very few/few clinical teaching workload, ‘thinking it is my obligation to carry out teaching work seriously’ and ‘thinking it is my duty to train medical talents’ were not only significant determinants of good perception but also good practice. Additionally, ‘thinking hospital attached great importance to clinical teaching’ was the significant determinant of good perception. *Insights*: Chinese clinical teachers demonstrate less positive perception and practice regarding the roles of teacher than clinical teachers in developed countries. Affiliated hospitals of medical universities should hold training sessions regularly and take targeted intervention measures to enhance clinical teachers’ perception and practice regarding the roles of teacher.

## Introduction

China education is the largest education system in the world. China’s education system is composed of four components, i.e., basic education, occupational/polytechnic education, higher education and adult education. Higher education comprises junior college, bachelor, master and doctoral degree programs, and medicine is an important component of higher education. Currently, medicine has completely blended into higher education, with everything that involves for the caliber of medical education (a University-based curriculum), and all students mix academic instruction with hands-on training at medical facilities [1]. In recent years, medical education has attracted more and more attention from the society in China, as it is related to both educational development and health, the two most concerned social issues for the people. On medical education, the National Health Commission of China and the Ministry of Education of China are taking moral education as the first task of medical talent cultivation, integrating ideological education and professionalism education throughout the entire process of education and teaching, further strengthening clinical practice skills training, so as to enhance the professionalism and clinical practice ability of medical students [2]. In terms of clinical teachers developments, the Ministry of Education of China and the National Health Commission of China have also proposed to further strengthen the training of physicians in hospitals and teachers in medical universities to improve their teaching abilities, and finally improve the quality of medical education [2]. The Lancet ‘Commission on Education of Health Professionals for the 21st Century’ published a widely acclaimed report in 2010 that called for a complete and authoritative re-examination of health professional education, and a new generation of medical education professionals who can better respond to current and future health challenges in order to improve human health [3]. With the rapid development of society and the deepening of the reform of higher medical education, medical education is transformed to a more student-centered, problem-based, and systematic approach from traditional teaching mode. Consequently, medical educators need to be updated to meet their new roles [4].

Clinical teaching is an important part of higher medical education system, which directly affected the quality of higher medical education and the quality of medical talent cultivation. Most of the clinical teaching tasks are mainly undertaken by affiliated hospitals of medical colleges and universities. For example, in the United Kingdom, more than 70% of clinical teaching and curriculum planning and assessment were undertaken by clinical staff in the National Health Service based teaching hospitals, district general hospitals and primary care [5,6]. Clinical teaching not only includes the teaching of theoretical courses, the guidance of internship courses, and the cultivation of internship stages, but also involves participating in the formulation of professional talent training plans, the compilation of teaching outlines, the compilation of teaching cases and textbooks, the implementation of assessment and evaluation, and the development of teaching reforms. At the same time, it also pays attention to the cultivation of humanistic qualities and assurance of good medical ethics among medical students. It ensures that clinical teachers, as the main bearers of clinical teaching, play a crucial role in medical education.

Differing from both university teachers and physicians, clinical teachers mainly work in hospitals, provide clinical services, administrative management, postgraduate or undergraduate student training and other work in addition to clinical teaching activities, and most clinical teachers have not participated in teaching ability training [5,7,8]. Clinical teachers face the daunting task of mastering the many domains of knowledge needed for practice and teaching, and the breadth and complexity of this knowledge continue to increase [9], as does the difficulty of transforming the knowledge into concepts that are understandable to learners [9]. Additionally, with the increase of the number of medical students and the growth of residents’ demand for medical and health services, the clinical workload and teaching workload of clinicians have greatly increased, which brings great working pressure to clinical teachers. Effective clinical teachers have been described in previous studies as clinically knowledgeable, compassionate, having strong integrity and possessing solid teaching skills [10]. The Association for Medical Education in Europe (AMEE) Guide No. 20 presented teachers’ twelve roles, such as a lecturer in a classroom setting, an information provider, role model on-the-job, curriculum evaluator, curriculum planner, course organizer, and so on [4,11]. To the best of our knowledge, in China, there is no published study analyzed the perception and practice of clinical teachers regarding the role of teachers. The purpose of this study, therefore, was to explore the perception of clinical teachers about their roles and current practice at affiliated hospitals of medical universities in China.

## Methods

### Study context

The study was conducted at seven affiliated hospitals of four major medical universities in Guangxi province, China. The seven affiliated hospitals were the First Affiliated Hospital of Guangxi Medical University (1^st^AFGXMU), the Second Affiliated Hospital of Guangxi Medical University (2^nd^AFGXMU), the Affiliated Cancer Hospital of Guangxi Medical University (ACHGXMU), the Affiliated Stomatology Hospital of Guangxi Medical University (ASHGXMU), the Affiliated Hospital of Youjiang Medical University for Nationalities (AHYJMUN), the First Affiliated Hospital of Guangxi University of Chinese Medicine (1^st^AFGXUCM), and the Affiliated Hospital of Guilin Medical University (AFGLMU). The target students of the clinical teachers were students majoring in clinical medicine and dentistry from medical university Guangxi Medical University, Youjiang Medical University for Nationalities, Guangxi University of Chinese Medicine, and Guilin Medical University. These four medical universities are managed by the Guangxi province Department of Education and have a unified education system. The clinical education projects and teaching contents of the four universities are uniformly planned and formulated by the Guangxi province Department of Education in accordance with the requirements of the Ministry of Education of China. Subsequently, the academic affairs office designs educational objectives and teaching requirements suitable for the development of students, based on the situation of university and students, while the hospital’s academic affairs department is responsible for organizing and implementing clinical teaching.

### Participants and data collection

A cross-sectional survey was administered from September 2022 to February 2023 at the seven affiliated hospitals. The target population was full-time physicians who were undertaking clinical teaching tasks. Participants were recruited using convenience sampling. Inclusion criteria required clinical teachers to be enrolled full-time, willing to participate in the study, and competent in providing informed consent. Exclusion criteria were part-time staffs, providing incomplete responses to the questionnaire, and those who did not participated in clinical teaching. This study was approved by the Ethics Committee of the Guangxi Medical University (No. KY20220138). All methods were carried out in accordance with relevant guidelines and regulations. A self-administered online questionnaire was used for data collection through ‘Wen Juan Xing’ (a professional online survey platform in China). The potential participants were all physicians who met the inclusion criteria. The link and Quick Response code of the online questionnaire were sent to the directors of various departments through the teaching management department of the hospital, and then forwarded to all physicians who were undertaking clinical teaching tasks. The clinical teachers independently completed questionnaires using their personal mobile phone, and the questionnaire could only be successfully submitted when it was completely filled out. Before the survey, all participants were informed that participation was voluntary, anonymous, and confidential. For those who did not complete the questionnaire, we would provide the list to the hospitals’ teaching management department to notify them to complete the survey.

### Survey

The survey included demographic information, the questionnaire of perception of importance regarding teacher roles, and the questionnaire of practice towards different roles. Before conducting the formal investigation, a small-scale preliminary survey was conducted in two hospitals (1^st^AFGXMU and 2^nd^AFGXMU) included in the seven affiliated hospitals through ‘Wen Juan Xing’, with a total of 32 physicians surveyed in both hospitals. And then, a small portion of the questionnaire was then revised by three experts, according to the results of the preliminary survey.

### Demographic information

A demographic questionnaire was developed to collect sociodemographic information. Participants were asked to provide the following personal information: gender (female, male), age (<30, 30–39, 40–49, ≥50), marital status (married, cohabitation, divorced, widowed, single), highest academic degree (bachelor’s degree, master’s degree, doctor degree), hospitals’ name, professional title (resident physician, attending physician, associate chief physician, chief physician), department, number of years engaged in clinical work (<10, 10–19, ≥20), number of years engaged in teaching work (<10, 10–19, ≥20), clinical teaching workload (very few, few, moderate, many, a great many), participated in teaching ability training (yes/no), devoted a lot of time to teaching work (yes/no), whether they thought the hospital attached great importance to clinical teaching (yes/no), whether they considered themselves as a clinician as well as a teacher (yes/no), whether they thought it is their obligation to carry out teaching work seriously (yes/no), whether they thought it is their duty to train medical talents (yes/no).

### Questionnaire of perception

The questionnaire of perception contained questions about the perception of importance regarding the role of teachers, which consisted of items extrapolated from AMEE Guide No. 20 [4,11]. Each item had the following response options on a 5-point Likert scale: Score 1=strongly disagree, 2=disagree, 3=neutral, 4=agree, 5=strongly agree. The total score ranged from 12 to 60 with higher scores indicating higher levels of perception. The median was used as the cut-off value. Regarding perception, the median was found to be 50, and those who scored < 50 were categorized as ‘poor’ and those scored ≥ 50 were categorized as ‘good’.

### Questionnaire of practice

The questionnaire of practice contained questions about the practice towards different roles of teacher, which also consisted of items extrapolated from AMEE Guide No. 20 [4,11]. Items were rated on a 5-point Likert scale that scored from 1 to 5 (1=never, 2=rarely, 3=sometimes, 4=frequently, 5=always). It was summed to obtain an overall score for a range of 12 to 60, where higher scores implied better practice. Regarding practice, the median value was 34, and those who scored < 34 were categorized as ‘poor’ and ‘good’ if the value was ≥ 34.

### Statistical analysis

The data were analyzed using the Statistical Package for the Social Sciences (SPSS) 22.0 (IBM Corp., Armonk, NY, USA). Descriptive statistics were computed for all study variables. Categorical variables were described by frequencies and percentages. A *chi*-square test was used to determine significant differences for categorical variables. A *p*-value < 0.05 was considered statistically significant. To avoid multicollinearity, 16 separate tests examined relationships between sociodemographic characteristics with perception/practice, controlling for age and gender. To control for Type I errors due to multiple tests, a Bonferonni correction was used to determine statistical significance; *p*-values < 0.003 (0.05/16) were considered significant. To further explore the influencing factors for perception/practice of clinical teachers regarding the role of teachers, variables with a *p*-values < 0.05 were included in multivariate binary logistic regression model.

## Results

### Sample characteristics

A total of 312 participants (178 females, 134 males) successfully completed the survey. The sociodemographic characteristics of the sample are presented in Table 1. Of the respondents, 178 (57.1%) were female, most of them (63.5%) aged 30 to 39 years old, 248 (79.5%) were married or cohabited, and 161 (51.6%) got a master's degree. Over a third (39.7%) of participants were attending physicians, 104 (33.3%) were associate chief physicians, and more than 45% engaged in clinical work or teaching work less 10 years, but 67.9% had participated in teaching ability training. Out of the 312 participants, 74 (23.7%) had many/a great many clinical teaching workload, and 192 (61.5%) devoted a lot of time to the clinical teaching work. More than 80.0% of the respondents responded positively to the statements of ‘I am a clinician as well as a teacher’, ‘it is my obligation to carry out teaching work seriously’, ‘it is my duty to train medical talents’ and ‘the hospital attached great importance to clinical teaching’.

**Table 1.** Sociodemographic characteristics of participants.

Variable	N	%
Gender		
Male	134	42.9
Female	178	57.1
Age		
<30	19	6.1
30–39	198	63.5
40–49	79	25.3
≥50	16	5.1
Marital status		
Married/cohabitation	248	79.5
Divorced/widowed/single	64	20.5
Degree		
Bachelor degree	42	13.5
Master degree	161	51.6
Doctor degree	109	34.9
Affiliated Hospitals		
1^st^AFGXMU	72	23.1
2^nd^AFGXMU	60	19.2
ACHGXMU	42	13.5
ASHGXMU	41	13.1
AHYJMUN	32	10.3
1^st^AFGXUCM	43	13.7
AFGLMU	22	7.1
Professional title		
Resident physician	50	16.1
Attending physician	124	39.7
Associate chief physician	104	33.3
Chief physician	34	10.9
Departments		
Internal medicine	70	22.4
Surgery	50	16.0
Gynecology and obstetrics	45	14.4
Pediatrics and neonatology	45	14.4
Medical imaging	47	15.2
Anesthesiology	30	9.6
Others	25	8.0
Years of engaged in clinical work		
<10	148	47.4
10–19	120	38.5
≥20	44	14.1
Years of engaged in teaching work		
<10	141	45.2
10–19	129	41.3
≥20	42	13.5
Clinical teaching workload		
Very few/few	125	40.1
Moderate	113	36.2
Many/a great many	74	23.7
Participated in teaching ability training		
Yes	212	67.9
No	100	32.1
I think the hospital attached great importance to clinical teaching		
Yes	251	80.4
No	61	19.6
I think I am a clinician as well as a teacher		
Yes	272	87.2
No	40	12.8
I think it is my obligation to carry out teaching work seriously		
Yes	269	86.2
No	43	13.8
I think it is my duty to train medical talents		
Yes	265	84.9
No	47	15.1
I devoted a lot of time to teaching work		
Yes	192	61.5
No	120	38.5

1^st^AFGXMU: the First Affiliated Hospital of Guangxi Medical University; 2^nd^AFGXMU: the Second Affiliated Hospital of Guangxi Medical University; ACHGXMU: the Affiliated Cancer Hospital of Guangxi Medical University; ASHGXMU:the Affiliated Stomatology Hospital of Guangxi Medical University; AHYJMUN: the Affiliated Hospital of Youjiang Medical University for Nationalities; 1^st^AFGXUCM: the First Affiliated Hospital of Guangxi University of Chinese Medicine; AFGLMU: the Affiliated Hospital of Guilin Medical University.

### Perception of clinical teachers regarding the role of teachers

The Cronbach’s alpha in this part of the questionnaire was 0.82. As shown in Table 2, the minimum mean score was 4.21 and the maximum mean score was 4.78, and the average score of perception was 4.51 (*SD* = 0.72). Additionally, most clinical teachers perceived that the teacher’s most important roles were working as a lecturer in a classroom setting (95.83%, scored 4.78 ± 0.54), teaching in clinical or practical class setting (93.27%, scored 4.62 ± 0.67), and on-the-job role model (92.95%, scored 4.66 ± 0.59). The least perceived roles were found to be the curriculum evaluator, role model in the teaching setting, and developing learning resource materials (84.94%, 88.46% and 89.74%, scored 4.21 ± 0.50, 4.57 ± 0.85, and 4.25 ± 0.85, respectively).

**Table 2.** Perception of clinical teachers regarding the role of teachers.

Teachers’ role	N (%)	Mean ± SD
Lecturer in classroom setting	299 (95.83)	4.78 ± 0.54
Teacher in clinical or practical class setting	291 (93.27)	4.62 ± 0.67
On-the-job role model	290 (92.95)	4.66 ± 0.59
Role model in the teaching setting	276 (88.46)	4.57 ± 0.85
Mentor, personal adviser or tutor to a student or group of students	285 (91.35)	4.50 ± 0.95
Learning facilitator, eg supporting students’ learning in problem based learning small groups in the laboratory, in the integrated practical class sessions or in the clinical setting	285 (91.35)	4.72 ± 0.63
Planning or participating in formal examinations of students	283 (90.71)	4.53 ± 0.80
Curriculum evaluator – evaluation of the teaching programme and the teachers	265 (84.94)	4.21 ± 0.50
Curriculum planner, participating in overall planning of the curriculum, through for example, curriculum planning committees such as the Undergraduate Medical Education Committee	282 (90.38)	4.25 ± 0.80
Course organizer, responsibility for planning and implementing a specific course within the curriculum. This may, for example, relate to one system or one theme, or to a special study module	286 (91.67)	4.45 ± 0.85
Production of study guides to support the students’ learning in the course	282 (90.38)	4.52 ± 0.69
Developing learning resource materials in the form of computer program, videotape or print which can be used as adjuncts to the lectures and other sessions	280 (89.74)	4.25 ± 0.85
Overall perception score	–	4.51 ± 0.72

SD: standard deviations.

### Practice of clinical teachers towards different roles

The Cronbach’s alpha in this part of the questionnaire was 0.85. As shown in Table 3, the minimum mean score was 3.25, the maximum mean score was 4.57, and the mean score of practice was 3.69 (*SD* = 1.17). Additionally, it was found that concerning the practice of clinical teachers towards the different roles, the top three highest practiced roles from clinical teachers were working as a lecturer in classroom setting (91.02%, scored 4.57 ± 0.70), teacher in clinical or practical class setting (81.41%, scored 3.88 ± 1.21), and learning facilitator (80.77%, scored 4.03 ± 1.26). Conversely, the least practiced roles were the production of study guides, the curriculum evaluator and developing learning resource materials (70.19%, 70.51% and 71.79%, scored 3.25 ± 1.16, 3.65 ± 0.86, and 3.38 ± 1.11, respectively).

**Table 3.** Practice of clinical teachers towards different roles.

Teachers’ role	N (%)	Mean ± SD
Lecturer in classroom setting	284 (91.02)	4.57 ± 0.70
Teacher in clinical or practical class setting	254 (81.41)	3.88 ± 1.21
On-the-job role model	232 (74.36)	3.76 ± 1.34
Role model in the teaching setting	236 (75.64)	3.69 ± 1.37
Mentor, personal adviser or tutor to a student or group of students	225 (72.12)	3.71 ± 1.30
Learning facilitator, eg supporting students’ learning in problem based learning small groups in the laboratory, in the integrated practical class sessions or in the clinical setting	252 (80.77)	4.03 ± 1.26
Planning or participating in formal examinations of students	228 (73.08)	3.51 ± 1.24
Curriculum evaluator – evaluation of the teaching programme and the teachers	220 (70.51)	3.65 ± 0.86
Curriculum planner, participating in overall planning of the curriculum, through for example, curriculum planning committees such as the Undergraduate Medical Education Committee	232 (74.36)	3.40 ± 0.91
Course organizer, responsibility for planning and implementing a specific course within the curriculum. This may, for example, relate to one system or one theme, or to a special study module.	229 (73.40)	3.52 ± 1.22
Production of study guides to support the students’ learning in the course	219 (70.19)	3.25 ± 1.16
Developing learning resource materials in the form of computer program, videotape or print which can be used as adjuncts to the lectures and other sessions	224 (71.79)	3.38 ± 1.11
Overall practice score	–	3.69 ± 1.17

SD: standard deviations.

### Category for perception and practice

Out of the 312 participants, 180 (57.69%) perception score of clinical teachers regarding the role of teachers was found to be good, but more than 40% as poor perception. Similarly, 168 (53.85%) participants have good practice towards the role of teacher, with nearly half of the participants as poor perception.

### Factors associated with participants’ perception and practice

As shown in Table 4, undertaking very few/few clinical teaching workload, ‘thinking the hospital attached great importance to clinical teaching’, ‘thinking it is my obligation to carry out teaching work seriously’, and ‘thinking it is my duty to train medical talents’ were found to have a significant association with the participants’ good perception regarding the role of teachers (all *p* < 0.003).

**Table 4.** Association of sociodemographic characteristics with perception and practice.

Variable	Score of perception		P-value	Score of practice		P-value
	Good N (%)	Poor N (%)		Good N (%)	Poor N (%)	
Gender			0.943			0.341
Male	77 (42.8)	57 (43.2)		68 (40.5)	66 (45.8)	
Female	103 (57.2)	75 (56.8)		100 (59.5)	78 (54.2)	
Age			0.889			0.172
<30	12 (6.7)	7 (5.3)		14 (8.3)	5 (3.5)	
30–39	114 (63.3)	84 (63.6)		102 (60.7)	96 (66.7)	
40–49	46 (25.6)	33 (25.0)		41 (24.4)	38 (26.3)	
≥50	8 (4.4)	8 (6.1)		11 (6.6)	5 (3.5)	
Marital status			0.760			0.119
Married/cohabitation	142 (78.9)	106 (80.3)		128 (76.2)	120 (83.3)	
Divorced/widowed/single	38 (21.1)	26 (19.7)		40 (23.8)	24 (16.7)	
Degree			0.109			0.813
Bachelor degree	21 (11.7)	21 (15.9)		23 (13.7)	19 (13.2)	
Master degree	102 (56.7)	59 (44.7)		89 (53.0)	72 (50.0)	
Doctor degree	57 (31.6)	52 (39.4)		56 (33.3)	53 (36.8)	
Affiliated Hospitals			0.480			0.135
1^st^AFGXMU	39 (21.7)	33 (25.0)		42 (25.0)	30 (20.8)	
2^nd^AFGXMU	40 (22.2)	20 (15.2)		33 (19.6)	27 (18.8)	
ACHGXMU	23 (12.8)	19 (14.4)		24 (14.3)	18 (12.5)	
ASHGXMU	23 (12.8)	18 (13.6)		24 (14.3)	17 (11.8)	
AHYJMUN	20 (11.1)	12 (9.1)		9 (5.4)	23 (16.0)	
1^st^AFGXUCM	26 (14.4)	17 (12.9)		24 (14.3)	19 (13.2)	
AFGLMU	9 (5.0)	13 (9.8)		12 (7.1)	10 (6.9)	
Professional title			0.170			<0.001
Resident physician	33 (18.3)	17 (12.9)		40 (23.8)	10 (6.9)	
Attending physician	68 (37.8)	56 (42.4)		53 (31.5)	71 (49.3)	
Associate chief physician	64 (35.6)	40 (30.3)		53 (31.5)	51 (35.4)	
Chief physician	15 (8.3)	19 (14.4)		22 (13.2)	12 (8.4)	
Departments			0.216			0.237
Internal medicine	47 (26.1)	23 (17.4)		40 (23.8)	30 (20.8)	
Surgery	27 (15.0)	23 (17.4)		27 (16.1)	23 (16.0)	
Gynecology and obstetrics	23 (12.8)	22 (16.7)		29 (17.3)	16 (11.1)	
Pediatrics and neonatology	23 (12.8)	22 (16.7)		23 (13.7)	22 (15.3)	
Medical imaging	24 (13.3)	23 (17.4)		22 (13.1)	25 (17.4)	
Anesthesiology	22 (12.2)	8 (6.1)		11 (6.5)	19 (13.1)	
Others	14 (7.8)	11 (8.3)		16 (9.5)	9 (6.3)	
Years of engaged in clinical work			0.446			0.510
<10	80 (44.4)	68 (51.5)		82 (48.8)	66 (45.8)	
10–19	74 (41.1)	46 (34.8)		60 (35.7)	60 (41.7)	
≥20	26 (14.5)	18 (13.7)		26 (15.5)	18 (12.5)	
Years of engaged in teaching work			0.017			0.017
<10	82 (45.6)	59 (44.7)		74 (44.0)	67 (46.5)	
10–19	66 (36.7)	63 (47.7)		63 (37.5)	66 (45.9)	
≥20	32 (17.7)	10 (7.6)		31 (18.5)	11 (7.6)	
Clinical teaching workload			0.002			<0.001
Very few/few	82 (45.6)	43 (32.6)		81 (48.2)	44 (30.6)	
Moderate	68 (37.7)	45 (34.1)		61 (36.3)	52 (36.1)	
Many/a great many	30 (16.7)	44 (33.3)		26 (15.5)	48 (33.3)	
Participated in teaching ability training			0.017			0.008
Yes	132 (73.3)	80 (60.6)		125 (74.4)	87 (60.4)	
No	48 (26.7)	52 (39.4)		43 (25.6)	57 (39.6)	
I think the hospital attached great importance to clinical teaching			<0.001			0.140
Yes	160 (88.9)	91 (68.9)		130 (77.4)	121 (84.0)	
No	20 (11.1)	41 (31.1)		38 (22.6)	23 (16.0)	
I think I am a clinician as well as a teacher			0.006			0.026
Yes	165 (91.7)	107 (81.1)		153 (91.1)	119 (82.6)	
No	15 (8.3)	25 (18.9)		15 (8.9)	25 (17.4)	
I think it is my obligation to carry out teaching work seriously			0.003			<0.001
Yes	164 (91.1)	105 (79.5)		158 (94.0)	111 (77.1)	
No	16 (8.9)	27 (20.5)		10 (6.0)	33 (22.9)	
I think it is my duty to train medical talents			<0.001			0.001
Yes	174 (96.7)	91 (68.9)		153 (91.1)	112 (77.8)	
No	6 (3.3)	41 (31.1)		15 (8.9)	32 (22.2)	
I devoted a lot of time to teaching work			0.089			0.542
Yes	118 (65.6)	74 (56.1)		106 (63.1)	86 (59.7)	
No	62 (34.4)	58 (43.9)		62 (36.9)	58 (40.3)	

1stAFGXMU: the First Affiliated Hospital of Guangxi Medical University; 2ndAFGXMU: the Second Affiliated Hospital of Guangxi Medical University; ACHGXMU: the Affiliated Cancer Hospital of Guangxi Medical University; ASHGXMU:the Affiliated Stomatology Hospital of Guangxi Medical University; AHYJMUN: the Affiliated Hospital of Youjiang Medical University for Nationalities; 1stAFGXUCM: the First Affiliated Hospital of Guangxi University of Chinese Medicine; AFGLMU: the Affiliated Hospital of Guilin Medical University.

On the other hand, with low professional title, undertaking very few/few clinical teaching workload, ‘thinking it is my obligation to carry out teaching work seriously’, and ‘thinking it is my duty to train medical talents’ were significantly associated with the participants’ good practice towards different roles (all *p* < 0.003) (Table 4).

### Multivariable binary logistic regression model for perception and practice

To explore the important influencing factors for perception/practice of clinical teachers regarding the role of teachers, a multivariable binary logistic regression model was conducted. Table 5 showed that undertaking very few/few clinical teaching workload (*AOR* = 0.657, *95% CI*: 0.368–0.811), ‘thinking the hospital attached great importance to clinical teaching’ (*AOR* = 1.894, *95% CI*: 2.782–5.667), ‘thinking it is my obligation to carry out teaching work seriously’ (*AOR* = 4.758, *95% CI*: 3.485–9.250), and ‘think it is my duty to train medical talents’ (*AOR* = 7.640, *95% CI*: 3.697–12.699) were significant determinants of good perception of clinical teachers regarding the role of teachers.

**Table 5.** Multivariable binary logistic regression model for perception and practice of clinical teachers.

Variables		*P*	*AOR*	95% *CI*
**Perception**				
	Clinical teaching workload	0.001	0.657	0.368-0.811
	I think the hospital attached great importance to clinical teaching	0.001	1.894	2.782-5.667
	I think it is my obligation to carry out teaching work seriously	0.003	4.758	3.485-9.250
	I think it is my duty to train medical talents	<0.001	7.640	3.697-12.699
**Practice**				
	Clinical teaching workload	<0.001	0.518	0.565-0.903
	I think it is my obligation to carry out teaching work seriously	0.003	3.601	2.004-6.348
	I think it is my duty to train medical talents	<0.001	2.557	1.869-3.563

Model was controlled for gender and age.

Additionally, undertaking very few/few clinical teaching workload (*AOR* = 0.518, *95% CI*: 0.565–0.903), ‘thinking it is my obligation to carry out teaching work seriously’ (*AOR* = 3.601, *95% CI*: 2.004–6.348), and ‘thinking it is my duty to train medical talents’ (*AOR* = 2.557, *95% CI*: 1.869–3.563) were significant determinants of good practice of clinical teachers towards different roles (Table 5).

## Discussion

A teacher is not only a role model who has an influence on every facet of students’ growth and on developing their innate potential but is also a motivator, guide, and friend. Association for Medical Education in Europe (AMEE) Guide no 20 describes the responsibilities of the teacher as 12 roles arranged in six dimensions, which has been widely used by teachers to evaluate their personal roles [12]. The present study showed that the average score of perception of clinical teachers regarding the role of teachers was at a high level (4.51 ± 0.72), which was similar to the study in Sudan (4.50 ± 0.14) [4], but significantly higher than that conducted in Pakistan (3.98 ± 0.25) [13]. What’s more, the results from our study indicated that clinical teachers in affiliated hospitals at Chinese medical schools hold an optimistic and positive attitude towards their teacher roles. Most of the participants (95.83%) perceived the most important role of the teacher as a lecturer in classroom setting, followed by a teacher in clinical or practical class setting (93.27%), and an on-the-job role model (92.95%). Similarly, a study in Sudan documented that the most important role as a lecturer at the classroom setting, followed by a teacher in clinical or practical class setting, and curriculum evaluator [4]. A study in Thailand revealed that three most highly rated roles were the clinical teacher, the on-the-job role model and the lecturer in classroom setting [14]. Nevertheless, in Saudi Arabia, the most important role perceived was that of an information provider in a clinical setting, followed by an on-job role model [15]. The least perceived roles in our study are found to be the curriculum evaluator, role model in the teaching setting, and learning resource developer, which is in accordance with the results of some previous studies [15,16]. Main factors that attributed to these differences were national policy, teaching strategy, the training of teachers, the educational environment, national cultures, and economic reasons. Thus, it can be seen, the role of a clinical teacher has extended beyond the boundaries of information providers, and recognized a clinical teacher to be not only an information provider but also an on-the-job role model to medical students in the process of teaching activities.

Concerning the practice of clinical teachers towards the different roles, the present study found that clinical teachers were highly committed to being a lecturer in the classroom setting (91.02%), followed by a teacher in clinical or practical class setting (81.41%) and learning facilitator (80.77%). This indicates that the practices are basically consistent with the perception of clinical teachers regarding the role of teachers, and clinical teachers carry out the teaching work according to their perceived teachers’ role. However, the result of the study reflects that clinical teaching is still conducted with a teacher-centered approach, although the education management department emphasizes the educational concept of ‘student-centered’, which is not conducive to cultivating students’ active learning ability. Additionally, the least practiced roles of clinical teachers were the production of study guides, the curriculum evaluator and learning resource developer, which was similar to the study conducted in Sudan [4]. However, what differs from the results of our study is that previous study emphasized the high commitment and practice of faculty members to other teacher roles, including curriculum developer, resource developer, learning facilitator, assessor and assessment creator, and leadership and managerial role [17]. These differences may be caused by national policies, teaching strategies, ethnic culture, and economic factors. Under the current education system of China, production of study guides, the curriculum evaluation and developing learning resources mainly designed jointly by experts in relevant fields, rather than the front-line clinical teachers.

Overall, senior faculty members have been engaged in clinical teaching for a long time and have accumulated a lot of teaching experience, which is more conducive to carrying out clinical teaching. Similarly, we found the clinical teacher engaged in clinical teaching ≥20 years had a better perception and better practice regarding the role of teachers. Previous study indicated that senior faculty members were more focused on roles with educational expertise, especially as an assessor and curriculum evaluator, whereas relatively less experienced teachers were attached more importance to traditional roles with medical expertise [15]. Nevertheless, our study showed that residents had a better practice of clinical teachers regarding the role of teachers, which may be due to the fact that residents in China are in the stage of professional development with accumulating experience, undertaking less teaching workload, so as to focus greater emphasis on the practice of clinical teachers regarding the role of teachers. Therefore, we further found that the clinical teacher undertaking fewer teaching workload had a better perception and practice regarding the role of teachers. Clinical teachers undertaking only few teaching workload was negatively associated with the occurrence of role conflict. Previous study revealed that high workload made physicians more likely believe their efforts was unappreciated and their patient relationships were inequitable, which increased physicians’ emotional exhaustion and work-family conflict [18].

Teaching training may improve the teaching level and teaching skills of clinical teachers, enhance their professional literacy, and promote their adaptation to the role of teachers [19,20]. An interdisciplinary study indicated that most resident doctors who participated in the teaching training expressed greater enthusiasm for teaching (both during and after residency), more learner centered approaches to teaching, and a much richer understanding of clinical teaching principles and skills [19]. Although the multivariable binary logistic regression analysis was not evaluated as a determinant of good perception/practice, univariate analysis indicated that participation in teaching training was closely related to good perception and good practice. The present study is completely consistent with the previous studies, which revealed the importance of education training programs in increasing the level of faculty member’s perception and practice regarding the role of teachers. At present, many hospitals have begun to launch the teaching training of clinical teachers gradually standardize and diversify. However, most of the teaching training are held in the way of training class or competition with small coverage and short time, so that the effect of the teaching training is not obvious. Therefore, we call on teaching management departments in hospitals to carry out regular and systematic professional training for clinical teachers, so as to improve the quality of clinical teaching.

The essence of emotional teaching was that teachers integrate emotions into teaching activities at students’ cognitive levels, realizing the teaching objective and improving the teaching effect [21]. Teaching emotion and teacher’s beliefs were important factors affecting the teaching quality. The more positive emotion and belief engaged in teaching, the better practice of clinical teachers regarding the role of teachers. Our study indicated that clinical teachers with the emotions and beliefs that ‘thinking I am a clinician as well as a teacher’, ‘thinking the hospital attached great importance to clinical teaching’, ‘thinking it is my obligation to carry out teaching work seriously’, and ‘thinking it is my duty to train medical talents’ had a better perception and better practice regarding the role of teachers. Hence, the hospital should attach great importance to clinical teaching and establish reasonable incentive mechanism to arouse the teaching emotion of clinical teachers, so as to improve the teaching quality and adapt to the teaching work better.

There are several limitations of this study that should be acknowledged. First, it is a cross-sectional study conducted in seven affiliated hospitals, which may not represents the perceptions and practice of all clinical teachers in China. Therefore, it is important to conduct a larger nationwide multi-centre study to obtain more scientific and accurate results. Secondly, all the data were collected based on self-report survey by an online questionnaire survey platform, without having any opportunity for participants to consult or explain any questions. Thirdly, clinical teachers responded to the online survey through the professional online survey platform in China, so we were unable to calculate the response rate [22].

## Conclusions

The present study examined the perception of clinical teachers about their roles and current practice at affiliated hospitals of medical universities in China. This study found that undertaking very few/few clinical teaching workload, ‘thinking it is my obligation to carry out teaching work seriously’ and ‘thinking it is my duty to train medical talents’ were not only significant determinants of good perception but also good practice of clinical teachers regarding the role of teachers. Additionally, ‘thinking hospital attached great importance to clinical teaching’ was the significant determinant of good perception. Nevertheless, the findings of the present study provided some initial insights to understand the perception and practice of clinical teachers and improve clinical teaching quality.
